# Low expression of miR-381 is a favorite prognosis factor and enhances the chemosensitivity of osteosarcoma

**DOI:** 10.18632/oncotarget.11861

**Published:** 2016-09-06

**Authors:** Yunchao Li, Chunhua Zhao, Zhibin Yu, Jiarui Chen, Xiaoling She, Peiyao Li, Changhong Liu, Yan Zhang, Jianbo Feng, Haijuan Fu, Bing Wang, Lei Kuang, Lei Li, Guohua Lv, Minghua Wu

**Affiliations:** ^1^ Department of Spinal Surgery, The Second Xiangya Hospital of Central South University, Changsha, Hunan, China; ^2^ Cancer Research Institute, School of Basic Medical Science, Central South University, Key Laboratory of Carcinogenesis and Cancer Invasion, Ministry of Education, Key Laboratory of Carcinogenesis, Ministry of Health, Changsha, Hunan, China; ^3^ Xiangya Nursing School, Central South University, Changsha, Hunan, China; ^4^ Pathology Department, The Second Xiangya Hospital of Central South University, Changsha, Hunan, China

**Keywords:** miR-381, osteosarcoma, LRRC4, chemosensitivity

## Abstract

Osteosarcoma (OS) is the most common primary bone malignancy with a poor prognosis for all races and both sexes. In this study, we found that miR-381 is a positive prognosis factor for OS patients that OS patients with a low expression of miR-381 had a longer survival time after surgical intervention, and miR-381 expression promotes MG-63 cell proliferation and cell invasion ability. Our results also showed a strong negative correlation between the expression of miR-381 and LRRC4 (brain relative specific expression gene) in OS tissues. This demonstrated that LRRC4 is a direct target gene of miR-381, and suppressing the expression of miR-381 increases the sensitivity of OS cells to chemotherapeutic drugs through the LRRC4-mediated mTOR pathway. In summary, miR-381 is an important biomarker in directing therapeutic intervention and predicting prognosis in OS patients.

## INTRODUCTION

Osteosarcoma (OS) is the most common primary bone malignancy and a contributor to the cancer mortality in children and adolescents. Osteosarcoma has a strong tendency to metastasize with a 10-20% prevalence of metastasis among OS patients, and most of the time it metastasizes to the pulmonary system [1]. In the last few decades, aggressive surgical resection combined with chemotherapy has improved the prognosis of patients with OS, but the prognosis remains poor. The 5-year survival rate for OS patients with localized disease is 65% [2], and the 5-years survival rate for recurrent OS and patients with pulmonary metastases is only 30% [1]. The genetic and molecular mechanisms of OS remain poorly understood. Thus it is crucial to find new targets for OS diagnosis and treatment in improving its overall prognosis.

MicroRNAs (miRNAs) are a large family of universally present small noncoding endogenous RNAs. In most situations, a single miRNA decreases the expression of a large number of genes by mRNA cleavage or translational repression [3]. Recent studies have demonstrated that miRNAs have significant functions in cellular differentiation, proliferation and apoptosis. There is also growing evidences that miRNAs can serve as biomarkers for cancer diagnosis and treatment. The expression of microRNA 381 (miR-381) is dysregulated in various cancer types, which suggested that miR-381 functions as oncogenic or tumor-suppressive miRNAs [4]. Some studies showed that the expression levels of miR-381 were downregulated in epithelial ovarian cancer [5, 6], hepatocellular carcinoma [7], renal carcinoma [8, 9], lung adenocarcinoma [10] and colon cancer [11], and an increased miR-381 expression inhibits the malignancy of these tumors. On the contrary, other studies indicated that the expression of miR-381 is upregulated in glioma [12] and pituitary tumors [13] and reducing miR-381 expression inhibited the proliferation and invasion of cells. However, the role of miR-381 in OS remains largely unknown.

In this study, we demonstrated the potential function of miR-381 in OS. With a high miR-381 expression profile in OS patients, a relative low expression could be used as a positive prognosis indicator. We also found that the brain relative specific expression gene LRRC4 which negatively correlates with miR-381 in OS tissues is a direct target gene of miR-381. Suppression of miR-381 expression increased the sensitivity of OS cells to cisplatin through the LRRC4-mediated mTOR signaling pathway.

## RESULTS

### miR-381 is a potential prognostic biomarker for OS patients

**Figure 1:**
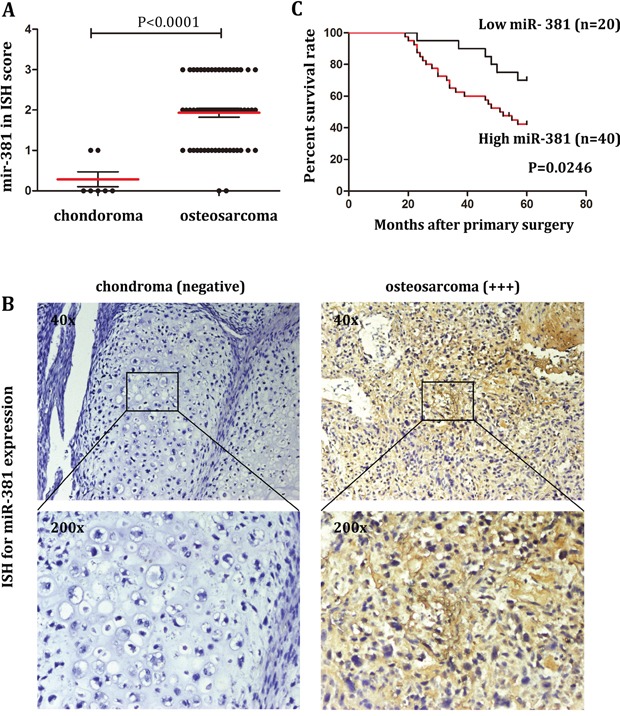
Upregulation of miR-381 in OS tissues is associated with poor prognosis **A.** ISH scores of miR-381 from 60 samples of 60 OS patients compared to 7 samples from 7 chondroma patients, as described in Materials and Methods. The data showed the miR-381 relatively high expression in OS. **B.** miR-381 expression in human chondroma and osteosarcoma determined by ISH. Images were obtained using 40x and 200x magnification. **C.** Kaplan-Meier analysis for overall survival in 60 OS patients in high- and low-risk groups based on miR-381 expression levels.

To detect miR-381 status in OS, in situ hybridization(ISH) was performed on paraffin sections of 60 samples from 60 OS patients and compared with 7 samples from 7 chondroma patients. As shown in Figure 1A and 1B, the expression of miR-381 is significantly higher in OS samples (n=60) than in chondroma samples (n=7). In the group of chondroma patients, 3 samples demonstrated negative miR-381 expression. On the contrary, only 2 samples had negative miR-381 expression which were scored as (-) in the group of OS patients, 18 samples demonstrated low miR-381 expression (+), 22 samples showed moderate miR-381 expression (++), and 18 samples presented high miR-381 expression (+++) (Figure 1A).

To determine the correlation between miR-381 expression and clinicopathologic characteristics of OS. We divided the OS patient into two groups according to the ISH score: high expression groups (n = 40) and low expression group (n = 20). As shown in Table 1, no statistical difference was demonstrated in the correlation between miR-381 expression and factors such as age, sex, localization nor metastasis. It is worth noting that miR-381 expression was statistically difference between postoperative recurrence group and non-postoperative recurrence group. Statistical analysis showed that the level of miR-381 expression was significantly associated with survival time (Table 1 and Figure 1C). Results from the Kaplan-Meier analysis also showed longer survival times for patients with relative lower miR-381expression by comparing with patient of high miR-381 expression. This demonstrates that miR-381 could potentially serve as a novel prognostic biomarker for OS patients.

**Table 1:** Correlation between the clinical factors and expression of miR-381 in OS (n=60)

Factors	n	miR-381 expression (%)		
		High	Low	P
Gender				
Male	37	24	13	0.783
Female	23	16	7	-
Age				
≤18	39	24	15	0.390
>18	21	16	5	-
location				
femur	36	22	14	0.402
other	24	18	6	-
Postoperative recurrence				
Yes	14	13	1	0.023*
No	46	27	19	-
Distant metastasis				
Yes	19	15	4	0.242
No	41	25	16	-

### miR-381 promotes MG-63 cell proliferation and invasion

**Figure 2:**
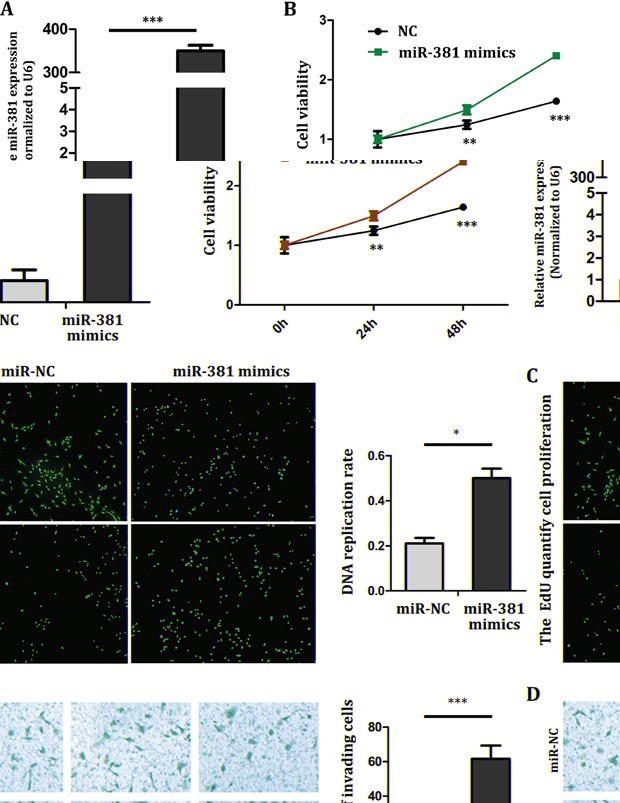
miR-381 overexpression promotes MG-63 cell proliferation and invasion **A.** RT–qPCR analysis of miR-381 expression in MG-63 was performed and normalized against an endogenous control (U6 RNA). **B**. CCK8 assay showed the increased proliferation of MG-63 cell by transfecting with miR-381 mimics. **C.** **left**: MiR-381 promoted the cellular DNA replication in MG-63. Images were obtained under a fluorescent microscope (magnification×40). The red fluorescence was diploid cells. **Right:** manual count the number of cellular DNA replication in MG-63, calculate the ratio. **D**. Transwell/Matrigel invasion assay showing overexpressed of miR-381 induced a marked increase in MG-63 cell invasion. * *p* <0.05; ** *p* <0.01; *** *p* <0.001.

In order to understand the role of miR-381 in the progression of OS, MG-63 cells were transfected with negative control (NC) and miR-381 mimics. RT-qPCR was used to determine the increase of miR-381 expression (Figure 2A). The Cell Counting Kit-8 (CCK8) assay showed that cells proliferation increased in MG-63 cells which were transfected with miR-381 mimics by comparing with NC group (Figure 2B). The 5-ethynyl-2-deoxyuridine (Edu) incorporation assay showed that ectopic miR-381 enhanced cellular DNA replication of MG-63 cells (Figure 2C). In addition, the Transwell/Matrigel assay results also showed that miR-381 overexpression induced a significant increase in cell invasion ability of MG-63 cells (Figure 2D). Hence, this indicates that miR-381 expression promotes MG-63 cell proliferation and cell invasion ability.

### miR-381 directly targets LRRC4 in MG-63 Cells

**Figure 3:**
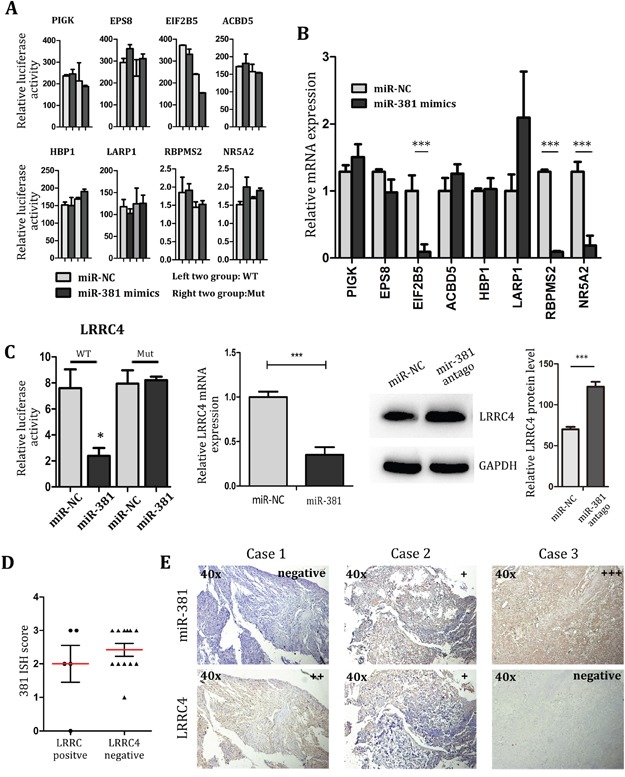
LRRC4 is target of miR-381, and LRRC4 suppresses MG-63 cell proliferation and invasion **A.** Luciferase assays of MG-63 cell co-transfected with pMIR-REPORT- WT/mutant 3′-UTR LRRC4 and miR-381 or the negative control. **B.** The real-time PCR showing the mRNA level of genes after transfected miR-381 mimics. **C.** **Left:** The luciferase, **Middle:** real-time PCR, **Right:** Western blot analysis all revealed that LRRC4 is target of miR-381 in OS. **D.** The score of 381 ISH in group of LRRC4 positive compared with the group of LRRC4 negative. **E.** The miR-381 and LRRC4 expression in same specimen. MiR-381 expression is negative correlation with LRRC4. * *p* <0.05; ** *p* <0.01; *** *p* <0.001.

As it was mentioned before, the biological function and target genes of miR-381 largely remains unknown. Here we predicted the PIGK, EPS8, EIF2B, ACBD5, HBP1, LARP1, RBPMS2, NR5A2 and LRRC4 to be the candidate target genes of miR-381 by using the miRanda, TargetScan and StarBase software. Luciferase assay and real-time PCR detection indicated that PIGK, EPS8, EIF2B, ACBD5, HBP1, LARP1, RBPMS2 and NR5A2 were not the direct target genes of miR-381(Figure 3A). Although real-time PCR suggested that EIF2B5, RBPMS2 and NR5A2 expression were suppressed by miR-381 in MG-63 cells, there was no statistical difference in its ability to modify all aspects of luciferase activity. So even miR-381could indirectly decrease the expression of RBPMS2 and NR5A2, it was unable to directly target the 3′-UTR of RBPMS2 and NR5A2. (Figure 3A, 3B). LRRC4 hence became the best candidate for direct target gene of miR-381 in MG-63 cells (Figure 3C).

We further investigated the LRRC4 expression in chondroma and OS samples. The immunohistochemistry analysis of LRRC4 expression in OS (n=24) demonstrated only positive results in 25% (n=6) of the samples, no statistically significant was found between OS and chondroma (Figure 3D). After comparing with results from the miR-381 ISH tested samples, it was noted positive LRRC4 expression was accompanied by a negative or low miR-381 expression in 5 positive LRRC4 cases. Conversely, in another 12 negative LRRC4 expression cases, negative LRRC4 expression was accompanied by moderate or high expression of miR-381. These results demonstrate an inverse relationship between expression of miR-381 and LRRC4 in OS samples (Figure 3E). The data hence suggest that the LRRC4 expression level could use as a good prognostic indicator for OS patients.

### miR-381 reversed the inhibition effect of LRRC4 on MG-63 cells

**Figure 4:**
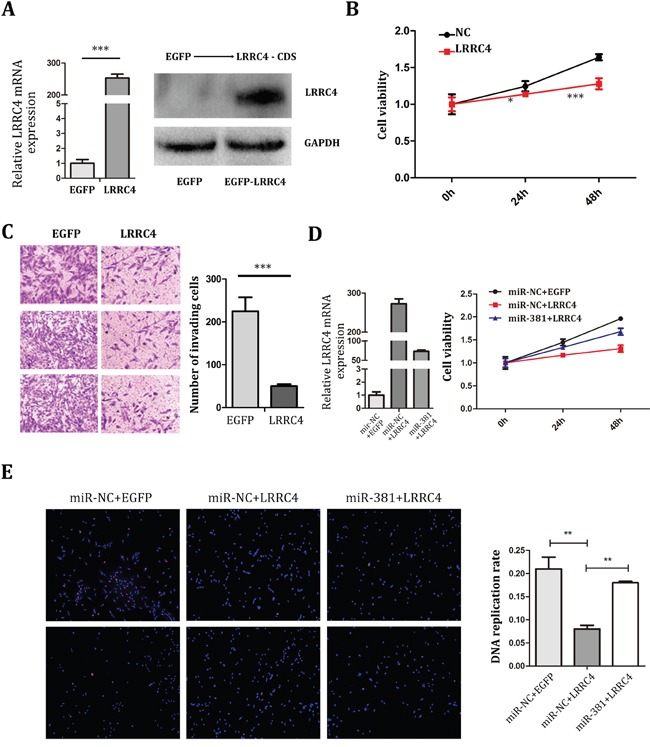
miR-381 Reversed the Inhibition Effect of LRRC4 on MG-63 Cells **A.** **Left:** The real-time PCR analysis of LRRC4 expression after transfected LRRC4, the LRRC4 obviously increased. **Right**: Western blot analysis revealed that after transcribed LRRC4 in MG-63 the protein expression of LRRC4 were increased. **B.** CCK8 verified that LRRC4 suppressed proliferation of MG-63 cell. **C.** Transwell/Matrigel invasion assay in MG-63 cell after LRRC4 overexpression. **D.** **Left**: The real-time PCR analysis of LRRC4 expression after transfected LRRC4 and recovered by 381 mimics. **Right:** CCK8 assay showed the miR-381 reversed the inhibition effect of LRRC4 on MG-63 cellular proliferation. **E.** EdU showed miR-381 reversed the inhibition effect of LRRC4 on MG-63 cellular DNA replication. * *p* <0.05; ** *p* <0.01; *** *p* <0.001.

To detect if LRRC4 functions as a tumor suppressor gene for OS, EGFP-LRRC4 plasmids were constructed and transfected into MG-63 cells (Figure 4A). LRRC4 was found to suppress the proliferation (Figure 4B) and invasion (Figure 4C) of MG-63 cells. miR-381 hence successfully reversed the inhibition effect of LRRC4 on MG-63 cell proliferation (Figure 4D) and cellular DNA replication (Figure 4E).

### miR-381 antago increased the chemotherapy sensitivity of MG-63 cells by targeting LRRC4

Chemotherapy plays an important role in comprehensive treatment of OS. In this study, 28 OS patients were given postoperative cisplatin chemotherapy. As shown in Figure 1C, OS patients who had a relative low miR-381 expression had a longer survival time. Thus, we investigated the cisplatin sensitivity in miR-381 knockdown MG-63 cells.

**Figure 5:**
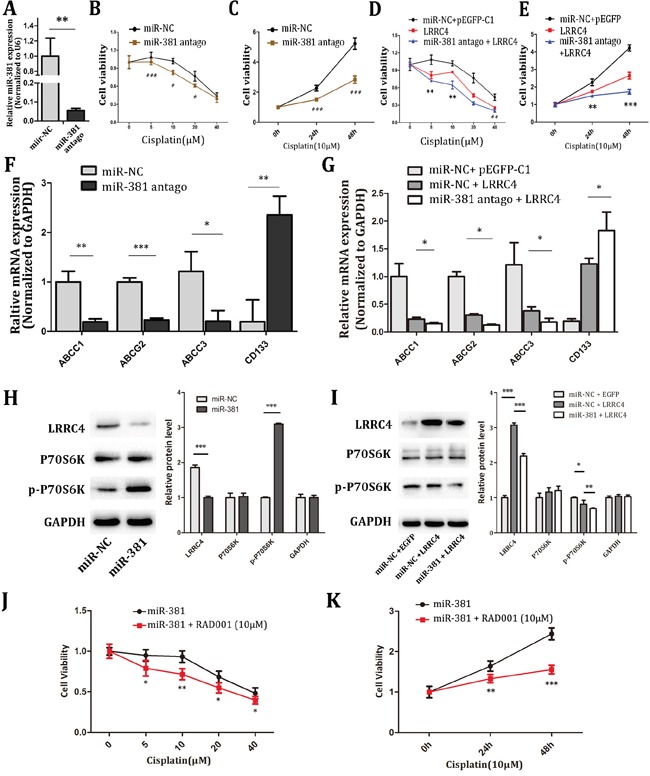
miR-381 antago increased the chemotherapy sensitivity of MG-63 cells by targeting LRRC4 transit mTOR pathway **A.** The real-time PCR showing the mRNA level of genes after transfected miR-381 antago. **B.** MG-63 cell transfected with NC or miR-381 antago were treated with various concentrations of cisplatin for 48 h and then subjected to CCK8 assays. **C.** MG-63 cell were transfected with miR-381 antago treated in cisplatin (10μM), then we tested the cellular activity in increasing time. **D, E.** CCK8 assays showed in MG-63, overexpressed LRRC4 improvement sensitivity to cisplatin. Inhibition of miR-381 could amplified LRRC4 sensibilization. **F.** The real-time PCR analyze expression of some multidrug resistance genes and D133 after transfected miR-381 antago in MG-63. **G.** LRRC4 suppressed the expression of these multidrug resistance genes and increased CD133. **H, I.** Western blot analysis the LRRC4, p-p70S6K and p70S6K expression. **J.** MG-63 cell after transfected miR-381 then incubated with RAD001 (10nM) for 24h, treated with diverse concentrations of cisplatin and detected cell activity 48h later. **K.** MG-63 cell after transfected miR-381 then incubated with RAD001 (10nM) for 24h, incubated with cisplatin (10μM) and detected the cellular activity in increasing time by using CCK8. * *p* <0.05; ** *p* <0.01; *** *p* <0.001.

After MG-63 cells were transfected by miR-381 antago (Figure 5A), pEGFP-LRRC4 plasmid or combination of miR-381 antago and pEGFP-LRRC4 plasmid for 24 hours, MG-63 cells were treated with different concentration of cisplatin, and cellular activity was measured by CCK8 assay at 48 hours. The data indicated that miR-381-antago enhanced the sensitivity of MG-63 cells to 10 μM cisplatin (Figure 5B and 5C). LRRC4 overexpression also enhanced the sensitivity of MG-63 cells to cisplatin, and the combination of miR-381 antago and LRRC4 has the most sensitivity effect (Figure 5D and 5E). Consequently, we investigated the effects of miR-381-antagomir on the multidrug resistance genes (ABCC1, ABCC3 and ABCG2) and CD133. miR-381-atagomir inhibited the expression of these multidrug resistance genes ABCC1, ABCC3 and ABCG2 but promoted CD133 expression (Figure 5F). Similar results were observed in MG-63 cells which were transfected with LRRC4. We also found that interfering with LRRC4 further changed the expression of these genes in MG-63 cells which were treated by miR-381 antago (Figure 5G). Further research indicated that miR-381 mimics increased phosphorylated p70S6K expression (Figure 5H), LRRC4 overexpression decreased phosphorylated p70S6K expression, and miR-381 reversed the LRRC4-mediated phosphorylated p70S6K expression (Figure 5I).

p70S6K, a critical downstream substrate, is used as a known downstream indicator of active mTOR pathway, we also tested the mTOR pathway inhibitor RAD001′s role on miR-381 pathway in the MG-63 cells. The cells were transfected with miR-381 mimics and cultivated with 10nM RAD001 for 24 hours, then treated with different concentrations of cisplatin, following by the CCK8 assay 48 hours later. It showed that mTOR inhibitor RAD001 reversed the relative inhibitory effect of miR-381 on cisplatin sensitivity in MG-63 cells (Figure 5J and 5K).

## DISCUSSION

Increasing reports have shown that miRNAs are important prognostic indicators and therapeutic targets for cancer. Differential expression of miR-381 had been shown to serve in different cancer types. For instance, miR-381 was downregulated in lung adenocarcinoma, and miR-381 involved in lung cancer cell migration and invasion [10]. miR-381 was found to be upregulated in glioma, suppression of miR-381 inhibited cellular proliferation of glioblastoma cells *in vitro* and growth of xenograft tumors *in vivo* [12, 14]. In this study, we found a high expression of miR-381 in OS compared to chondroma, miR-381 was associated with the prognosis of OS patients (low expression of miR-381 was a positive prognostic indicator). A brain-relative special expression gene - LRRC4 was identified as a direct target of miR-381 in OS cells. While miR-381 and LRRC4 are both negatively expressed in chondroma, it was surprising that their expression shared an inverse relationship in the OS. The data indicates that the expression of LRRC4 is negatively regulated by miR-381 in OS, with miR-381 acting as an onco-miRNA and LRRC4 functioning as a tumor suppressor gene. miR-381 fulfills its function by reversing the inhibition of MG-63 cells proliferation and DNA replication through LRRC4.

LRRC4 is a member of the LRRC4/NGL (netrin-G ligand) family. LRRC4/NGL-2 plays important role in the development of nerve system through regulating neurite outgrowth, lamina-specific dendritic segmentation, and axon differentiation. In addition, the overexpression of LRRC4 suppresses glioma cell growth, angiogenesis and invasion through complicated signaling regulation networks. LRRC4 also has the ability to form multiphase loops with miRNA, transcription factors and gene methylation modification factors. The loss of LRRC4 function hence may play important role in the biological processes of gliomas. In summary, LRRC4 is a critical gene in the normal development and tumorigenesis of the nervous system [15]. We speculate that despite being a brain tissue relative specificity expressing gene, LRRC4 was expressed in OS because of possible mesoderm origin [16].

Chemotherapeutic drugs, such as cisplatin, doxorubicin, and methotrexate, are important/commonly used in the treatment of OS. In this study, 28 OS patients were given postoperative cisplatin chemotherapy. We found OS patients with a relative low miR-381 expression had a better chemotherapy sensitivity and a longer survival time, however, OS patients who had high miR-381 expression had a poor chemotherapy sensitivity and a shorter survival time. Our research indicated that either knockdown miR-381 or LRRC4 overexpression enhanced the chemotherapy sensitivity of OS cells. By combining the knockdown of miR-381 and overexpressing LRRC4 together, better chemotherapy sensitivity was observed in OS cells than knock downing miR-381 or overexpressing LRRC4 alone.

Tumor cell drug resistance is an intractable common issue in chemotherapy and it is also the main cause of tumor recurrence and metastasis. In recent years, researchers have found a phenomenon that is some cancer cells which are resistant to certain chemotherapy drugs are also resistant to other drugs that are different in structure and mechanism. This phenomenon known as multidrug resistance (MDR). Cancer stem cells (CSCs) are a small subpopulation of tumor cells, which are similar to stem cells, and may be derived from stem cell transformation or non-stem cell differentiation [17–19]. CSCs have the ability of self-renewal and can differentiate into all of tumor cells. Researches show that some characteristics of CSCs such as quiescence, increased the efflux ability of drugs, increased the ability of DNA repair, and improved the ability to resist apoptosis contribute to the resistance of CSCs to oncotherapy, including radiation and chemotherapy [18, 20]. Therefore, even if radiotherapy and chemotherapy can significantly reduce the bulk of the tumor, it is still very difficult to accurately target the chief culprit - CSCs. CD133 is recognized as a marker to identify and isolate CSCs [21]. Multiple studies proved that CD133 high expression have closely related with tumor metastasis and poor prognosis in various cancers [22–25], even in OS [24].

In addition to CSCs, MDR most commonly results from the active adenosine triphosphate (ATP)-dependent transport of drugs out of the cell by efflux pumps belonging to the ATP-binding cassette (ABC) family of transporters [26, 27]. The ABC efflux transporters such as ABCC1 and ABCC3 are known as multidrug resistance-associated proteins. ABCG2 is known as breast cancer resistance protein can regulate the traffic of small molecules across the cell membrane [28], and Walters, DK [28, 29] showed there different expression of ABCG2 have some correlation between increasing chemoresistance in MG-63 cell line. In this study, we designed experiment to find miR-381 effect MDR and CSCs. We also found that either Inhibition miR-381 or overexpression LRRC4 decreased the expression of ABCC1, ABCC3, ABCG2 and upregulated the expression of CD133.

The mammalian target of rapamycin (mTOR) is an atypical serine/threonine protein kinase that belongs to the phosphoinositide 3-kinase (PI3K)-related kinase family [30] and interacts with several proteins to form two distinct complexes named mTOR complex1 (mTORC1) and 2 (mTORC2) [31]. mTOR has emerged as a critical effector in cell-signaling pathways commonly deregulated in human disorders, including cancers. For the crucial role of mTOR in cancer cell biology, Several mTOR inhibitors have already undergone clinical trials for treating tumors, and the first identified inhibitor, rapamycin (from which mTOR derives its name), are currently in clinical development [32–34]. It demonstrated that mTORC1 activation alone can cause carcinomas [35] Rapamycin is a highly selective mTORC1 inhibitor to suppress the tumor, but in numerous clinical trials, rapamycin gave only modest and lmited clinical benefits in patients with TS who had angiomyolipomas due to the solubility and pharmacokinetic properties [34]. The literature pointed that combination of the mTOR inhibitor sirolimus with cisplatin significantly increased cell death rate compared to either drug alone in four of 12 hMPM cell lines [33]. In this paper, we revealed that LRRC4 can improve the sensitivity of MG-63 cell to cisplatin when combined treatment with mTOR inhibitor. It could because LRRC4 improves the cisplatin sensitivity by inhibiting the mTOR pathway.

In conclusion, our study indicates that miR-381 functions as an oncogene, and LRRC4 serves as a tumour suppressor gene in OS. miR-381 directly downregulating LRRC4 expression. Low expression of miR-381 is a good prognosis indicator in patients with OS, and low expression of miR-381 is associated with higher cisplatin sensitivity. Suppressing miR-381 expression could increase the chemotherapy sensitivity in OS by targeting LRRC4. The distinct functions and properties of miR-381 have made it an important target of treating OS. Further patient cohort study is still needed to verify miR-381 expression in chemotherapy planning.

## MATERIALS AND METHODS

### Human tissue paraffin sections

Human osteosarcoma and chondroma paraffin sections were obtained from the Department of Pathology, The Second Xiangya Hospital, Central South University, Hunan, China. This study was approved by the Ethics Committee of The Second Xiangya Hospital, School of Central South University. All the paraffin sections were stored in the 4 °C until immunohistochemistry or in-situ hybridization (ISH) was performed.

### ISH and immunohistochemistry analyses

For ISH and Immunohistochemistry analysis, tissue sections were deparaffinized in xylene and hydrated in alcohol. The sections for ISH were then boiled in citrate solution for 5 minutes. Using the miR-381-ISH kit from Boster (Wuhan, Hubei, China) and according to the instruction manual, ISH was performed. The hsa-miR-381-LNA sequence 5′-3′ was 5′-ACAGAGAGCTTGCCCTTGTATA-3′. The other sections for Immunohistochemistry were microwaved for 20 minutes. Primary LRRC4 antibody (Santa Cruz, CA, USA) was manually applied at 1:100 dilution, and the sections were incubated at 4°C for 12 hours. The DAB detection kit was used for both the ISH and Immunohistochemistry tests to identify the presence of gene expression and the sections were counterstained with hematoxylin for 2 minutes. According to the area of staining, scores on an ordinary scale of 0-3 were given separately for the ISH and Immunohistochemistry tests. When <5%, 25-50%, 50-75% and >75% of the sections were stained, a score of 0, 1, 2, and 3 were given respectively. The scores from both tests were then added. If the added score was 0-1, 2-3, 4-5, or 6-7, the section received a final grade of negative (-), low (+), moderate (++), and high (+++) expression respectively [36]. Scoring for ISH and Immunohistochemistry was performed by two independent observers and were averaged.

### Cell culture

Human osteosarcoma cell lines MG-63 were cultured in Dulbecco's modified Eagle's medium (DMEM) (HyClone, Logan, UT) supplemented with 10% fetal bovine serum. Cells were incubated at 37 °C in a humidified atmosphere of 5% CO2 in air.

### Quantitative real-time PCR (qRT-PCR)

Total RNA was extracted from cell lines specimens using TRIzol reagent according to the manufacturer's instructions (Invitrogen, Carlsbad, USA). Realtime PCR reactions were performed using SYBR Premix Ex Taq II (Takara, Dalian, China) and human GAPDH or U6 snRNA was respectively used as an endogenous control for mRNA or miRNA detection. Expression of each gene was quantified by measuring Ct values and normalized using the 2-ΔΔct method relative to U6 snRNA or GAPDH. The primers used were all purchased from BGI·Tech (Shenzheng, China) and shown in Supplementary Table 1.

### Cell viability and edu assays

Cell viability was evaluated using CCK8 (Beyotime, Jiangsu, China). Cells (2 × 10^3^) were seeded in 96-well plates by different treatment, and cultured for different time points. CCK8 solution (10 μl) was added to each well, and the plates were incubated at 37°C for 1 hour. Absorbance was measured at 450 nm on a microplate reader (Bioteck). DMEM containing 10% CCK8 was used as a control. Proliferating cell count was measured using the Cell-Light EdU DNA Cell Proliferation Kit (RIBOBio Co, Guangzhou, China). The cells were seeded in 96-well culture plates and exposed to media with or without plumbagin. 2000 cells/well were treated with 50 μmol/L of EdU for 2 h at 37 °C. After being fixed with 4% paraformaldehyde for 15 min, the cells were baptized with 0.5% Triton X-100 for 30 minutes and rinsed with PBS three times. Thereafter, the cells were exposed to 100 μL/well of 1×Apollo® reaction cocktail for 30 minutes and incubated with 5 μg/mL of Hoechst 33342 to stain the cell nuclei for 30 minutes. Images were captured using a fluorescent microscope (Olympus, Tokyo, Japan). Experiments were repeated at least three times.

### Matrigel chamber invasion assay

Invasion assay was determined using 24-well BD Matrigel invasion chambers (Corning Inc., Corning, NY) in accordance with the manufacturer's instructions. 4×104 cells per well were seeded in DMEM with 10% serum in the upper well of the invasion chamber, and the lower chamber well contained DMEM with 20% FBS to stimulate cell invasion. After 24 hours, cells that invaded the lower chamber were fixed with 4% paraformaldehyde, stained with 0.1% crystal violet, and photographed in three independent fields for each well. Three independent experiments were conducted in triplicate.

### *In vitro* chemotheraphy sensitivity assay

The chemotheraphy sensitivity of the cell was determined by the CCK8 assay. Cells were seeded and transfected with miRNA or Plasmid using the Lipofectamine 3000(Invitrogen) transfection agent. After 24 hours, the cells were reseeded in 96-well plates at a density of 2,000 cells per well and treated with cisplatin (0-40μM) for 48 hours. Cell survival was analyzed using the CKK8 assay. Data was collected from three separate experiments with five replications performed each time.

### Western blotting

The protein was extracted by lysing cells in RIPA buffer containing protease and phosphatase inhibitors. The protein concentration was determined by using BCA assay (Thermo, USA). Aliquots of protein lysates were fractionated by SDS-PAGE, transferred to a PVDF membrane (Merck, Millipore, Germany), and subjected to immunoblotting analysis according to the manufacturer's instruction. ECL Detection System (Merck Millipore, Germany) was used for signal detection.

### Statistical analysis

Each experiment was repeated at least three times. All the data was analyzed with GraphPad Prism 5 (La Jolla, CA, USA). The Kaplan-Meier method was used to calculate the survival curve. The correlation between the clinical characteristics such as … and expression of miR-381 or LRRC4 was analyzed using the Fisher exact test. The correlation between miR-381 expression and LRRC4 levels in tumor tissues was analyzed using the Wilcoxon signed-rank test. Mean differences between the variables of the groups were tested using the Student's t-test or two-way ANOVA, using the SPSS 19.0 program. A *p*-value of <0.05 was considered to indicate a statistically significant result.

## SUPPLEMENTARY MATERIALS TABLE


