# Supplementary material for: Low expression of miR-381 is a favorite prognosis factor and enhances the chemosensitivity of osteosarcoma
Source: Oncotarget. 2016 Sep 6;7(42):68585–96. doi: 10.18632/oncotarget.11861 (PMC5356575; doi:10.18632/oncotarget.11861)
Supplement: Supplementary file 1 [file oncotarget-07-68585-s001.pdf]

Low expression of miR-381 is a favorite prognosis factor and enhances the chemosensitivity of osteosarcoma

SUPPLEMENTARY TABLE

Supplementary Table S1: The primers for miR-381, the predicted target genes, multidrug resistance factors and stemness facters

Gene name	Forward / Reverse primer(5′-3′)
PIGK	F:5′-GGCCGCTAGTCATATCGAGG-3′<br>R:5′-GTACACACCAGAACAGCCCA-3′
EPS8	F:5′-GCTGGAGTCGACCAGAACTC-3′<br>R:5′-GTTGGCTCTTTGCACACCAG-3′
EIF2B5	F:5′-CGACGATACTTGCAGACCGA-3′<br>R:5′-ATCACGGAGGACATCTCCCA-3′
ACBD5	F:5′-GCCTTGTCCGGCAATACCAA-3′<br>R:5′-CGGCAAACTCTGGATCACCT-3′
HBP1	F:5′-AATGGCGACGGGTTTGTCAG-3′<br>R:5′-TCCTGAGGCTCTCTTGTCCA-3′
LARP1	F:5′-CCTGGTCACTCCATGCTTTG-3′<br>R:5′-AATGGCTTCTCCCTGTCACC-3′
RBPMS2	F:5′-TCTCTGCCCGTTCTGTTCAC-3′<br>R:5′-ATGCCAGACACAAAGGCGTA-3′
NR5A2	F:5′-AGGGCGATCTCGAGTGTCT-3′<br>R:5′-TGGTCAGGTCAGAGGGCATA-3′
ABCC1	F:5′-GCCGATGGCTCCGACC-3′<br>R:5′-CGCATCCACCTTGGAACTCT-3′
ABCG2	F:5′-GAACCCAAGGAGATAGGAGA-3′<br>R:5′-CTAGACAGACTTCAACCAGG-3′
ABCC3	F:5′-CCTTCCAGGTAAAGCAAATG -3<br>R:5′-GTGTCAGGGTAGAGTCCAAT -3
CD133	F:5′-ATACCTAGGTCCCCGTCCG-3′<br>R:5′-TCCTGAAAAGGAGTTCCCGC-3′
GAPDH	F:5′-ATCAAGATCATTGCTCCTCCTGAG -3′<br>R:5′-CTGCTTGCTGATCCACATCTG -3′
U6	F: 5′-ATTGGAACGATACAGAGAAGATT -3′<br>R: 5′-GGAACGCTTCACGAATTTG -3′
miR-381	R: 5′-TGTCTCTCGAACGGGAACATAT-3′
